# Diversity and geographic distribution of haplotypes of *Dirofilaria immitis* across European endemic countries

**DOI:** 10.1186/s13071-023-05945-4

**Published:** 2023-09-12

**Authors:** Mustafa Alsarraf, Elena Carretón, Lavinia Ciuca, Anastasia Diakou, Dorota Dwużnik-Szarek, Hans-Peter Fuehrer, Marco Genchi, Angela Monica Ionică, Agnieszka Kloch, Laura Helen Kramer, Andrei D. Mihalca, Martina Miterpáková, Rodrigo Morchón, Elias Papadopoulos, Mateusz Pękacz, Laura Rinaldi, Mohammed Alsarraf, Mariia Topolnytska, Alice Vismarra, Anna Zawistowska-Deniziak, Anna Bajer

**Affiliations:** 1 Department of Eco-Epidemiology of Parasitic Diseases, Institute of Developmental Biology and Biomedical Sciences, Faculty of Biology, University of Warsaw, Miecznikowa 1, 02-096 Warsaw, Poland; 2 Internal Medicine, Faculty of Veterinary Medicine, University of Las Palmas de Gran Canaria, Campus Arucas, Arucas, 35413 Las Palmas, Spain; 3 Department of Veterinary Medicine and Animal Production, University of Napoli Federico II, Via Delpino 1, 80137 Naples, Italy; 4 Laboratory of Parasitology and Parasitic Diseases, School of Veterinary Medicine, Faculty of Health Sciences, Aristotle University of Thessaloniki, 54124 Thessaloniki, Greece; 5 Institute of Parasitology, University of Veterinary Medicine, Veterinaerplatz 1, 1210 Vienna, Austria; 6 Department of Veterinary Science, Parasitology Unit, University of Parma, strada del Taglio, 10, 43126 Parma, Italy; 7 Department of Parasitology and Parasitic Diseases, University of Agricultural Sciences and Veterinary Medicine of Cluj-Napoca, Calea Manastur 3-5, 400372 Cluj-Napoca, Romania; 8 Institute of Functional Biology and Ecology, Faculty of Biology, University of Warsaw, Miecznikowa 1, 02-096 Warsaw, Poland; 9 Institute of Parasitology, Slovak Academy of Sciences, Hlinkova 3, 040 01 Košice, Slovakia; 10 Zoonotic Diseases and One Health Group, IBSAL-CIETUS (Biomedical Research Institute of Salamanca-Research Centre for Tropical Diseases University of Salamanca), Faculty of Pharmacy, University of Salamanca, 37007 Salamanca, Spain; 11 Division of Parasitology, Department of Preclinical Sciences, Faculty of Veterinary Medicine, Warsaw University of Life Sciences-SGGW, Warsaw, Poland; 12 Department of Parasitology, Institute of Functional Biology and Ecology, Faculty of Biology, University of Warsaw, Miecznikowa 1, 02-096 Warsaw, Poland; 13 Department of Immunology, Institute of Functional Biology and Ecology, Faculty of Biology, University of Warsaw, Miecznikowa 1, 02-096 Warsaw, Poland

**Keywords:** Heartworm, Dogs, Spain, Greece, Hungary, Romania, Portugal, Slovakia, Ukraine, Italy, Bangladesh

## Abstract

**Background:**

*Dirofilaria immitis*, also known as heartworm, is one of the most important parasitic nematodes of domestic dogs, causing a potentially serious disease, cardiopulmonary dirofilariosis, which can be lethal. This species seems to be less 'expansive' than its sister species *Dirofilaria repens*, and it is believed that climate change facilitates the spread of this parasite to new non-endemic regions.

**Methods:**

In total, 122 heartworm isolates were analysed from nine endemic countries in Europe (Portugal, Spain, Italy, Greece, Hungary, Romania, Slovakia, and Ukraine) and a single isolate from Bangladesh by amplification and sequencing of two mitochondrial (mt) DNA markers: cytochrome c oxidase subunit 1 (COI) and dehydrogenase subunit 1 (NADH). The main aim of the current study was to determine the genetic diversity of *D. immitis* and compare it with *D. repens* haplotype diversity and distribution. DNA was extracted from adult heartworms or microfilariae in blood. Most isolates originated from dogs (*Canis lupus familiaris*) while 10 isolates originated from wildlife species from Romania, including eight isolates from golden jackals (*Canis aureus*), one isolate from a Eurasian otter (*Lutra lutra*) and one isolate from a red fox (*Vulpes vulpes*).

**Results:**

Median spanning network analysis was based on the combined sequence (1721 bp) obtained from two mt markers and successfully delineated nine haplotypes (Di1-Di9). Haplotype Di1 was the dominant haplotype encompassing 91 out of the 122 sequences (75%) from all nine countries and four host species. Haplotype Di2 was the second most common haplotype, formed solely by 13 isolates from Italy. The remaining sequences were assigned to Di3-Di9 haplotypes, differing by 1–4 SNPs from the dominant Di1 haplotype. There was evidence for geographical segregation of haplotypes, with three unique haplotypes associated with Italy and four others associated with certain countries (Di4 and Di7 with Slovakia; Di8 with Greece; Di6 with Hungary).

**Conclusion:**

Diversity in *D. immitis* mt haplotypes was lower by half than in *D. repens* (9 vs. 18 haplotypes in *D. immitis* and *D. repens*, respectively), which may be associated with the slower expansion of heartworm in Central and NE Europe. NADH gene appears to be conserved in *Dirofilaria* sp. by showing lower genetic diversity than the analysed COI gene.

**Graphical Abstract:**

**Figure:**
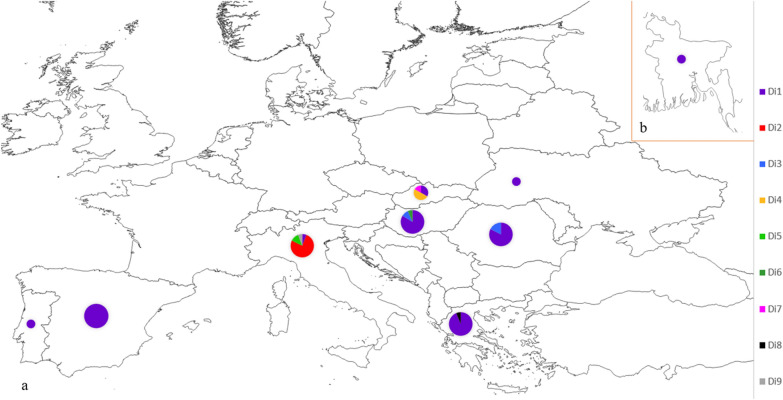

**Supplementary Information:**

The online version contains supplementary material available at 10.1186/s13071-023-05945-4.

## Background

Nematodes of the genus *Dirofilaria* are parasites vectored by mosquitoes, and domestic dogs constitute their main hosts and reservoir [1–4]. *Dirofilaria immitis*, the canine heartworm, causes cardiopulmonary dirofilariosis, also known as heartworm disease, in canids and can be fatal [5].

The adult worms are found in the pulmonary arteries where females produce microfilariae that are released into the circulatory system; when the worm burden increases, worms are also located in the right cardiac chambers [6–8].

The nematodes can also infect humans and cause human dirofilariosis because of the mosquito vectors feeding on infected dogs and later on humans [9, 10]. *Dirofilaria immitis* infections in humans can cause a wide variety of symptoms including chest pain, cough, obstructive pulmonary disorder and emphysema through the formation of pulmonary nodules in the lungs [11–13].

Many countries around the world are considered endemic for *D. immitis*, such as Spain, Greece, Hungary, Romania, Italy, Tunisia, Saudi Arabia, the USA, Mexico and Brazil [14–25].

However, the parasite is still not endemic in certain countries in Europe such as Poland, Czechia, Denmark, Finland, Iceland and Sweden, where either low or zero prevalence was reported [23, 25–31].

Climate change and increased dog travelling are considered the primary drivers of *D. immitis* expansion into new countries, especially from countries with a Mediterranean climate to countries with a continental climate, by providing suitable conditions for the development and survival of larval stages in the mosquito vectors [32–34].

Previous studies have shown that the cytochrome c oxidase I (COI) gene and the dehydrogenase subunit I (NADH) gene are competent barcoding mitochondrial (mt DNA) markers [35–37]. These markers were also used in a similar study which delineated 18 haplotypes of *Dirofilaria repens* [38]; therefore, these mt DNA markers were chosen for the molecular analysis of the isolates.

The main aims of this study were: (i) to evaluate the genetic diversity in *D. immitis* isolates from southern, central and eastern Europe and (ii) to compare the genetic diversity between *D. repens* and *D. immitis*. Investigating the diversity and geographic distribution of *D. immitis* haplotypes will allow future studies to track and predict the future expansion of *D. immitis* into new non-endemic regions (countries).

We hypothesize that genetic variation is present in *D. immitis* isolates from different geographically distant countries and manifests by geographical segregation of haplotypes.

## Methods

### Sample collection of *D. immitis*

In total, 122 heartworm isolates from different hosts were analysed from nine endemic countries, in Europe (Portugal, Spain, Italy, Greece, Hungary, Romania, Slovakia and Ukraine) and a single isolate from Bangladesh (Table 1) [39–42]. A single isolate was collected from a dog from Portugal which travelled with its owners and was diagnosed with *D. immitis* upon its arrival to Poland; therefore, it is safe to assume that the dog was infected in Portugal. Genomic DNA was extracted from blood isolates of microfilariemic dogs stored in EDTA (*n* = 66) or adult worms obtained during necropsies of different hosts and stored in 70% ethanol (*n* = 56) (Additional file 1: Dataset S1) using DNeasy Blood & Tissue kit (Qiagen, USA).

**Table 1:** Origin of *Dirofilaria immitis* isolates for molecular typing by country and region

Country	Spain	Portugal	Italy	Greece	Hungary	Romania	Slovakia	Western Ukraine	Bangladesh	Total
No. of isolates	25	1	17	16	31	23	7	1	1	122
Region (total)	Southern Europe (*n* = 59)				Central Europe (*n* = 61)			Eastern Europe	Southern Asia	

Most isolates (*N* = 112) were obtained from canine hosts representing all countries (Additional file 1: Dataset S1) while 10 isolates originated from wildlife species from Romania, including eight isolates from golden jackal (*Canis aureus*), one sample from Eurasian otter (*Lutra lutra*) and one sample from red fox (*Vulpes vulpes*).

### Molecular typing of *D. immitis*

Genotyping of *D. immitis* was performed by PCR amplification and sequencing of two mitochondrial genes (mt DNA): the cytochrome c oxidase subunit I (COI, 2 fragments [38]) and dehydrogenase subunit I (NADH) as described previously for *D. repens* study [38].

The first fragment of COI gene (849 bp) was amplified using the primers Drep2F and Ctc1R; the second fragment of COI gene (801 bp) was amplified using the primers Ctc2F and Ctc2R. The second mt marker, NADH gene fragment (516 bp), was amplified using the primers Nad1F and Nad1R. All PCR reactions were conducted in conditions and with thermal profiles described previously [38].

Amplicons were purified and sequenced in both directions (Sanger sequencing) by Genomed (Genomed S.A., Warsaw, Poland) and Eurofins Genomics (Ebersberg, Germany GmbH).

Both reads were checked for quality and then aligned and edited to form a consensus sequence using CodonCode Aligner 10.0.2 (CodonCode Corp., Centerville, MA, USA).

Consensus sequences from both (mt) DNA markers (COI: 1323 bp; NADH: 398 bp) were joined and the final 1721-bp-long sequence of (mt) DNA aligned using ClustalW in MEGA X 10.1.8.

DnaSP 6.12.03 [43] was used to calculate the number of haplotypes in the final (mt) DNA construct. Median spanning network analysis was performed for the (mt) DNA sequence using PopART 1.7 [44], and only sequences obtained in this study were used. Following no observed diversity in NADH gene and exploring usefulness of COI locus for haplotype delineation, a separate median spanning network analysis was repeated including only combined fragments of the COI gene (1323 bp). A map showing the haplotype geographic distribution by country was created using QGIS 3.24.

Sequence diversity was also calculated between the two species *D. immitis* and *D. repens* [38]. The tests included nucleotide diversity per site (*π*), haplotype diversity (Hd), the number of variable sites (S) and average number of nucleotide differences (K) using DnaSP 6.12.03 [43].

Obtained sequences were deposited in the GenBank, the first fragment of COI gene under the accession numbers OQ726801-OQ726922, the second fragment of COI gene under the accession numbers OQ726923-OQ727044 and the NADH gene under the accession numbers OQ736780-OQ736901 (Additional file 1: Dataset S1).

Phylogenetic trees were computed using maximum likelihood for both fragments of mt DNA marker COI and for the (mt) DNA marker NADH separately since all sequences obtained from the GenBank were too short in length to be analysed with the combined COI sequences. The evolutionary model (Tamura-Nei model) was chosen based on the model tested by Mega X [45] and bootstrapped over 1000 randomly generated sample trees with uniform rates among sites. Identical consensus sequences obtained in the study were pooled for analysis and accompanied by reference sequences obtained from the GenBank database. Appropriate regions of complete (mt) DNA genome of *D. repens* (KX265049) were used as an outgroup for the trees.

## Results

**Fig. 1:**
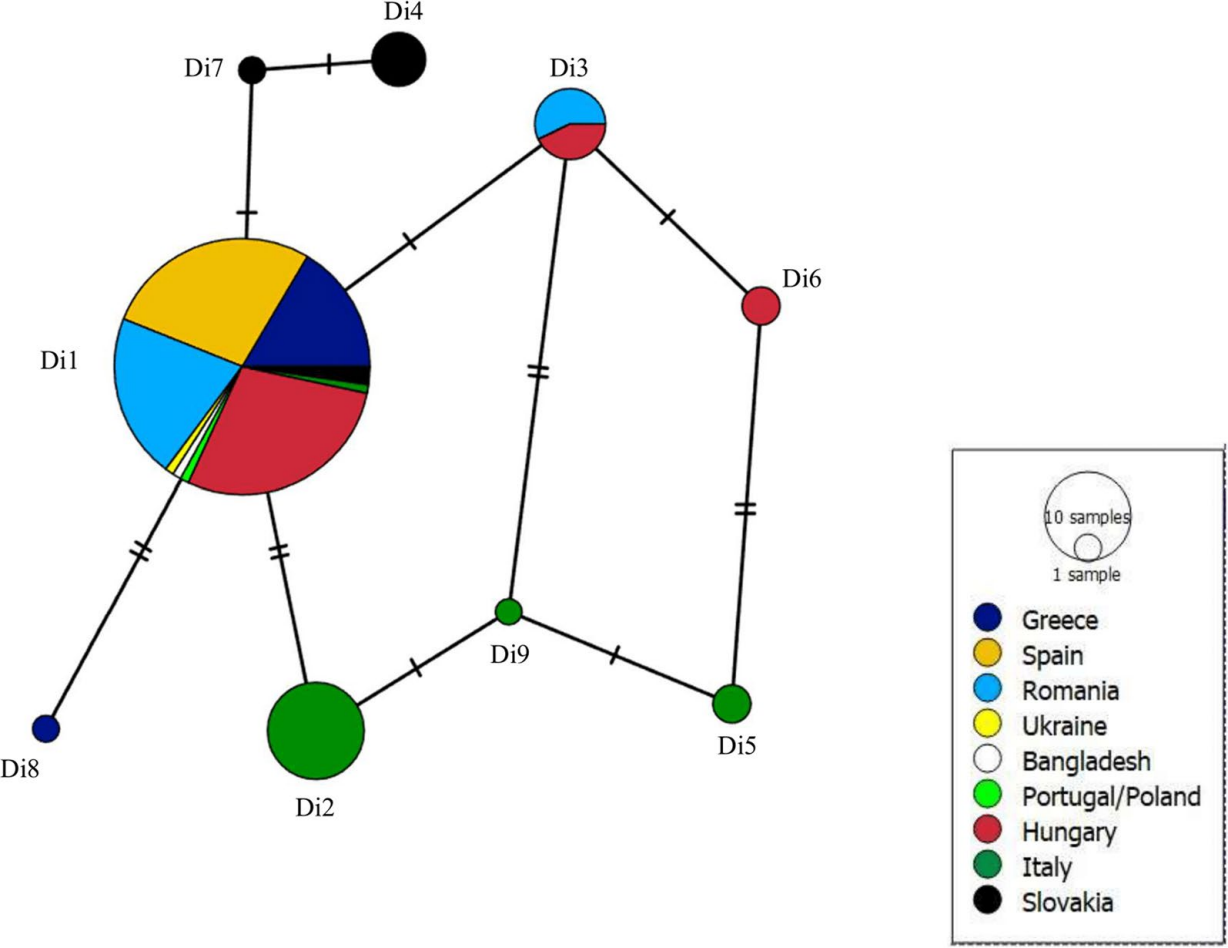
Median spanning network of mt DNA sequence (COI and NADH) showing a relationship between nine delineated haplotypes of *Dirofilaria immitis*

Nine haplotypes (Di1–Di9) were identified in the mt DNA among the 122 analysed isolates (Additional file 1: Dataset S1: database with isolates and accession numbers for genetic markers). Haplotype Di1 was the dominant haplotype encompassing 91 of the 122 sequences (75%), from all nine countries and four host species (Fig. 1; Additional file 1: Dataset S1). Haplotype Di2, differing by two single nucleotide polymorphisms (SNP) from the main haplotype Di1, was the second most common haplotype, formed solely by 13 isolates from Italy (13/122 = 11%). Haplotype Di3 encompassed seven sequences (6%), four from Romania and three from Hungary, with one SNP difference from the main haplotype Di1. All other haplotypes grouped around haplotype Di1 are separated by 1–4 SNPs (Fig. 1).

**Fig. 2:**
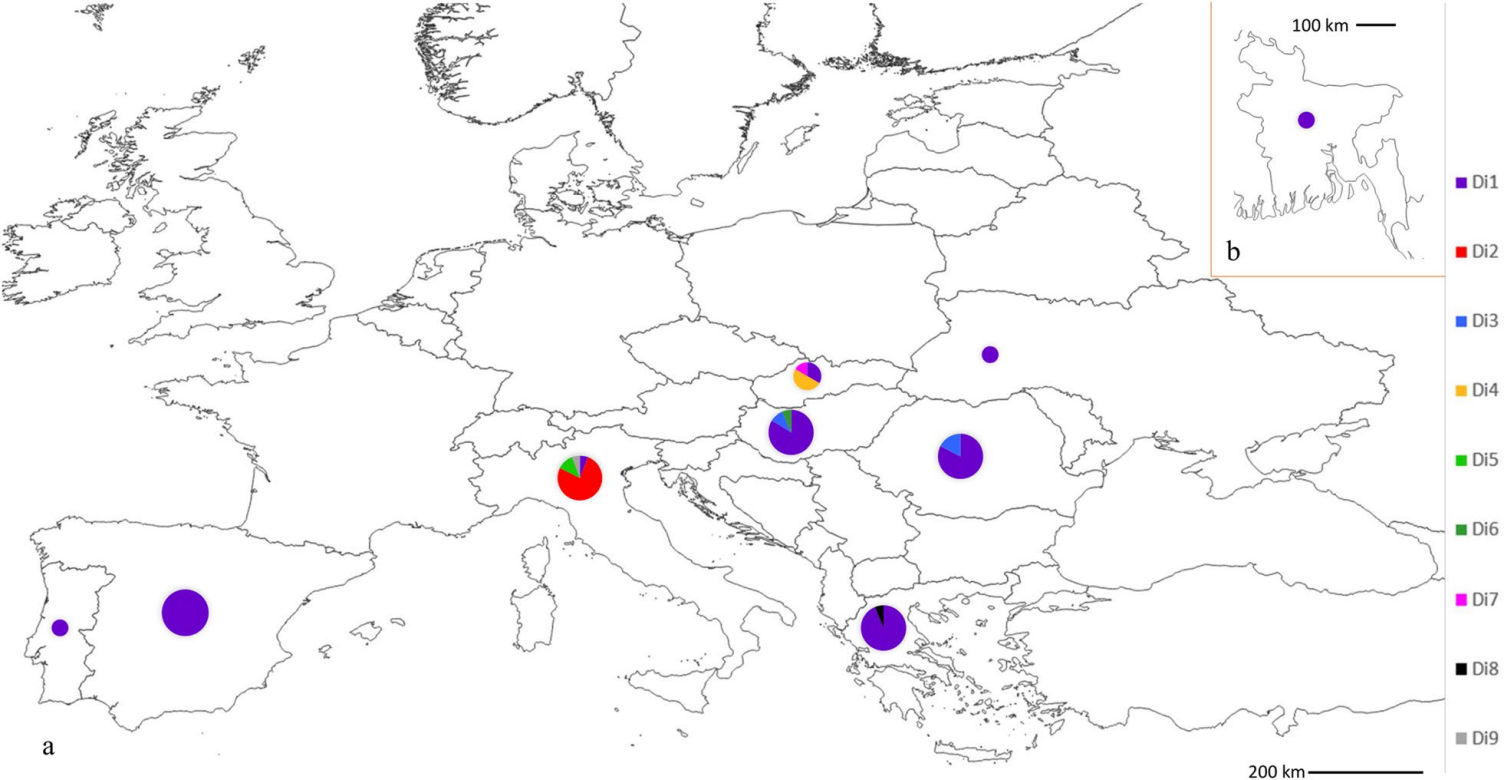
Geographic distribution of haplotypes by country of origin: **a** Haplotype distribution in eight European countries; **b** haplotype Di1 in Bangladesh

Despite this generally low genetic diversity, there was some geographical segregation of less common haplotypes (Figs. 1, 2). Interestingly, most *D. immitis* sequences from Italy (16/17) differed from sequences from other countries in southern Europe and formed three unique haplotypes (Di2, Di5 and Di9) (Figs. 1, 2). Four other unique haplotypes were associated with certain countries (Di4 and Di7 with Slovakia; Di8 with Greece; Di6 with Hungary) (Additional file 1: Dataset S1, Fig. 1).

Figure 2 shows the distribution of haplotypes by country of origin. Haplotype Di1 was present/dominant in all countries endemic for *D. immitis*, in southern and central Europe, with sporadic occurrence in the single isolates from Western Ukraine (Eastern Europe) and Bangladesh (South Asia). Interestingly, other haplotypes were dominant among *D. immitis* sequences from Italy and Slovakia (Fig. 2).

Another interesting result was that isolates obtained from microfilaraemic blood appeared to have higher genetic diversity than isolates from adult nematodes.

The most common haplotype Di1 was identified in 36 of 91 MF samples. Seven haplotypes were distinguished among the remaining 55 samples of MF, and only one more haplotype, Di8, was delineated in isolate from an adult worm from Greece. Furthermore, the median spanning network including only both fragments of the COI gene was identical to the network acquired from combined COI and NADH genes.

The comparison of the genetic diversity between *D. immitis* and *D. repens* showed that the measures of polymorphism (number of variable sites, S) in *D. immitis* were half those in *D. repens*, while the average number of nucleotide differences (K) and the nucleotide diversity (π) were similar in both species with a slight difference in haplotype diversity (Hd) between both species (Table 2).

**Table 2:** Diversity in the studied species *Dirofilaria immitis* and *D. repens*: N represents the number of studied sequences, *H* represents the number of haplotypes, *S* represents the number of variable sites, Hd represents the haplotype diversity, *K* represents the average number of nucleotide differences while *π* represents the nucleotide diversity per site

Species	Length (bp)	*N*	*H*	*S*	Hd	*K*	*π* × 10^3^
*D. immitis*	1721	122	9	7	0.431	0.874	0.51
*D. repens*	1520	95	18	15	0.528	0.887	0.58

**Fig. 3:**
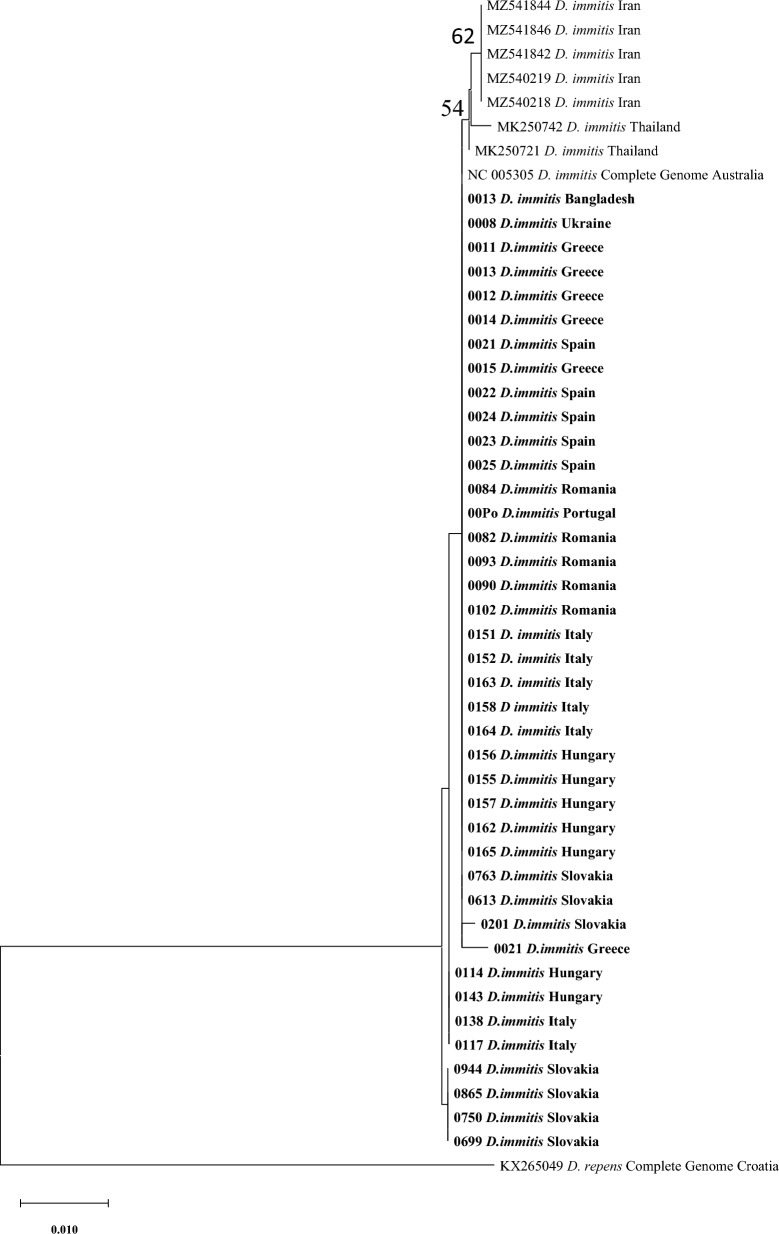
The evolutionary history was inferred by using the maximum likelihood method and Tamura 3-parameter model for the first fragment of COI (only bootstrap value above 50 is displayed in the figure). The tree with the highest log likelihood (− 1213.39) is shown. The percentage of trees in which the associated taxa clustered together is shown next to the branches. Initial tree(s) for the heuristic search were obtained automatically by applying neighbour-joining and BioNJ algorithms to a matrix of pairwise distances estimated using the Tamura 3 parameter model and then selecting the topology with superior log likelihood value. The tree is drawn to scale, with branch lengths measured in the number of substitutions per site. This analysis involved 49 nucleotide sequences. Codon positions included were 1st + 2nd + 3rd + Noncoding. A total of 671 positions were in the final dataset. Evolutionary analyses were conducted in MEGA X [46]

**Fig. 4:**
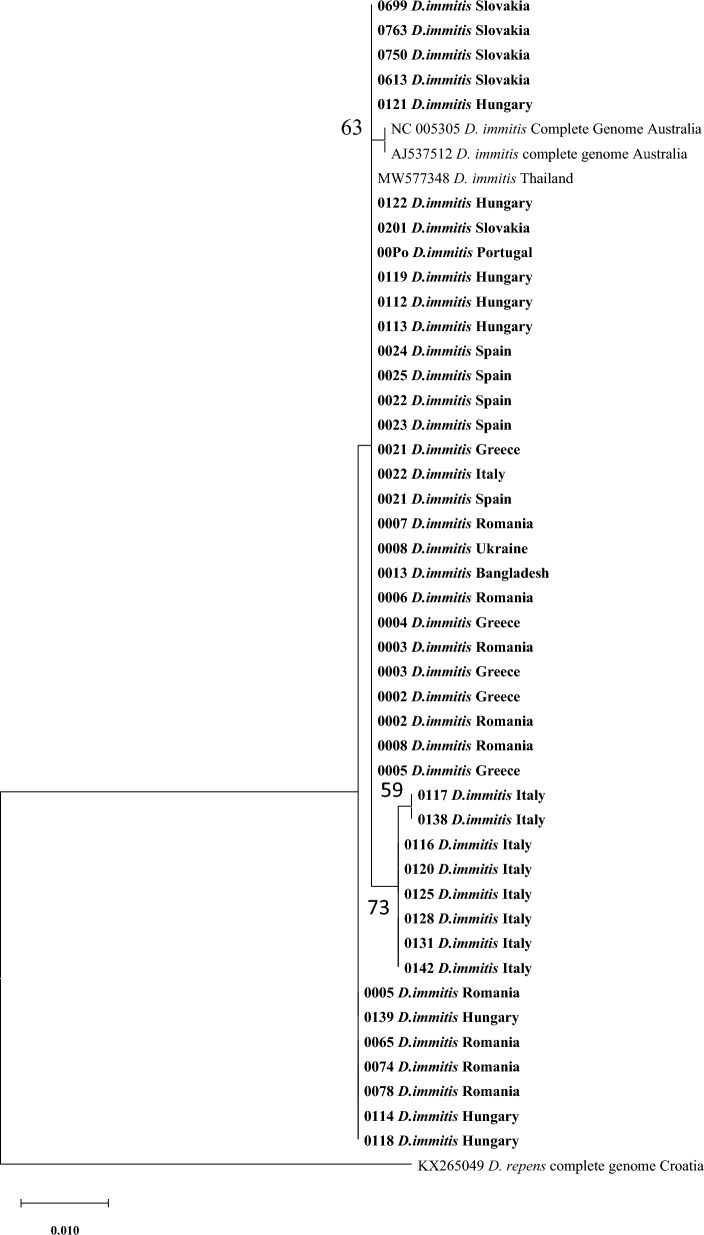
The evolutionary history was inferred by using the maximum likelihood method and Tamura 3-parameter model for the second fragment of COI (only bootstrap values > 50 are displayed in the figure). The tree with the highest log likelihood (− 1115.50) is shown. The percentage of trees in which the associated taxa clustered together is shown next to the branches. Initial tree(s) for the heuristic search were obtained automatically by applying neighbour-joining and BioNJ algorithms to a matrix of pairwise distances estimated using the Tamura 3 parameter model and then selecting the topology with superior log likelihood value. The tree is drawn to scale, with branch lengths measured in the number of substitutions per site. This analysis involved 48 nucleotide sequences. Codon positions included were 1st + 2nd + 3rd + Noncoding. A total of 649 positions were in the final dataset. Evolutionary analyses were conducted in MEGA X [46]

**Fig. 5:**
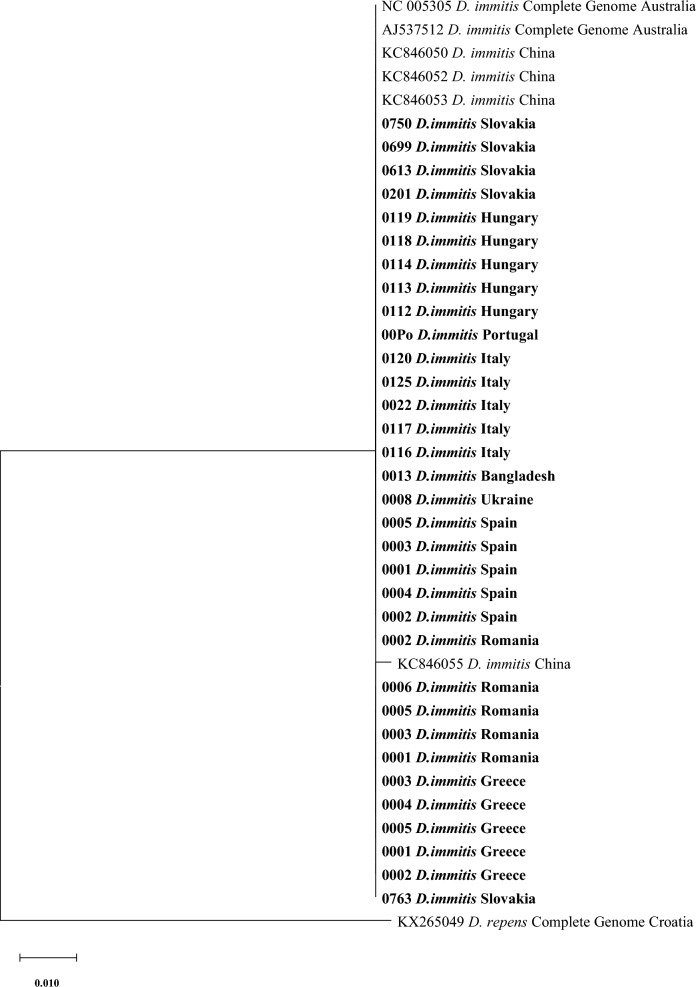
The evolutionary history was inferred by using the maximum likelihood method and Tamura three-parameter model for the full-length NADH gene (only bootstrap values > 50 are displayed in the figure). The tree with the highest log likelihood (− 632.78) is shown. The percentage of trees in which the associated taxa clustered together is shown next to the branches. Initial tree(s) for the heuristic search were obtained automatically by applying neighbour-joining and BioNJ algorithms to a matrix of pairwise distances estimated using the Tamura 3 parameter model and then selecting the topology with superior log likelihood value. The tree is drawn to scale, with branch lengths measured in the number of substitutions per site. This analysis involved 40 nucleotide sequences. Codon positions included were 1st + 2nd + 3rd + Noncoding. A total of 358 positions were in the final dataset. Evolutionary analyses were conducted in MEGA X [46]

For the comparison of our new data with sequences deposited previously in GenBank, especially regarding the sequences from the countries not represented in our examination, the phylogenetic analysis was conducted for each (mt) DNA marker/fragment separately (two fragments of COI and the full length NADH gene; Figs. 3, 4, 5).

The phylogenetic tree for the first fragment of COI gene (671 bp) is presented in Fig. 3. The tree shows that the majority of sequences from Greece, Spain, Bangladesh, Ukraine, Portugal, Romania, Italy, Hungary and Slovakia were closely related to sequences obtained from Iran, Thailand, Australia, Greece and Slovakia with a bootstrap value < 70. Four other sequences, two each from Hungary and Italy, grouped together, while four sequences from Slovakia grouped separately (Fig. 3).

The second phylogenetic tree included the analysis for the second fragment of COI (mt) DNA gene (649 bp), which shows that most sequences from all nine countries appear to be closely related to sequences from Hungary, Romania, Australia and Thailand with a bootstrap value < 70, while another branch included sequences only from Italy, which appear to be distinct from others with a bootstrap value of 75 (Fig. 4).

The third and final phylogenetic tree for the full NADH mt DNA gene (358 bp) showed no diversity; all sequences from this study grouped together to form a major branch joined by sequences from the GenBank from Australia and China (Fig. 5).

## Discussion

In the present study, the haplotype diversity in mt DNA genotyping genes (COI and NADH) of canine heartworms, *D. immitis*, in central and southern Europe was analysed. Combined (mt) DNA markers (COI + NADH) allowed the delineation of nine haplotypes and revealed some evidence for geographical segregation of haplotypes, despite the generally low genetic diversity and dominance of haplotype Di1 in dogs and other host species from the majority of countries.

The use of identical markers (COI + NADH) in the current and previous studies also allowed a comparison of haplotype diversity, structure and distribution between both *Dirofilaria* species occurring in Europe [38].

Most sequences (75%) formed the Di1 haplotype, which is in agreement with a previous study using mt sequences of COI and 12S rDNA derived from the GenBank, which showed that 272 out of the 277 *D. immitis* sequences (98%) formed a dominant haplotype [47]. In total, only five haplotypes were delineated based on these two mt markers from sequences from 26 countries [47], which is much lower than in the current study. One of the reasons could be that the haplotypes in the current study were based on the entirety of the combined COI and NADH genes, which offers a broader insight into the genetic diversity of each isolate and better delineation of haplotypes while the sequences used in previous study [47] covered a fragment of the COI gene. However, it is also evident from our repeated analysis that a long enough COI fragment may be used solely for haplotype delineation in *D. immitis,* because of no diversity in NADH gene.

Interestingly, the single *D. immitis* sequence from Bangladesh was in the main haplotype Di1, which may suggest that the Di1 haplotype may be the main haplotype occurring globally rather than just being dominant only in Europe. However, to confirm this point, more *D. immitis* sequences from Bangladesh and other non-European countries are needed. Spain and Greece are both southern European countries known to be endemic for *D. immitis* [14, 20, 25]; therefore, having all Spanish sequences and most of the Greek sequences included in the main haplotype Di1 was expected.

Hungary and Romania are neighbouring central European countries which are also endemic for *D. immitis* [21, 48]; both shared a unique haplotype (Di3) including seven sequences while being closely related to another unique haplotype including two Hungarian sequences (Di6).

Haplotype Di6 presents a possible link between the unique Italian haplotype (Di5) and the most common haplotype (Di1), which can be interpreted into geographic relation/similarity between the genetic diversity of isolates from Hungary and Italy.

Interestingly, a much lower diversity in (mt) DNA in *D. immitis* compared to *D. repens* was documented, which may be associated with the faster spread of *D. repens* in central and NE Europe [23, 49]. We hypothesized that the spread of *Dirofilaria* spp. to new environments (countries) and new biotic conditions (different species of hosts) can be facilitated by the genetic adaptation of the parasite to certain conditions, and this adaptation can take place by the occurrence of many genetic variants (high genetic diversity), which can be crucial for the survival and success of the parasite in different conditions. If so, more genetically ‘conserved’ parasites (*D. immitis* in this case) cannot be that successful in colonisation of different areas than more ‘flexible’ or genetically diverse species, like *D. repens*.

Regardless, a counter argument may also be true: a loss of genetic diversity can accompany the spread of the parasite species to a new area, with the success of a single genetic lineage/variant. Other lineages could be eliminated during expansion by negative selection by unfavourable conditions or just low number of breeding worms in expanding population. The evidence of this low genetic diversity was previously observed [50] in the raccoon roundworm (*Baylisascaris procyonis*), which infects the invasive raccoons (*Procyon lotor*) in Germany (expansion area). Two parasite lineages resulting from independent introductions of the parasite in Germany showed low genetic diversity suggesting small founding populations subjected to inbreeding and genetic drift.

In addition, in both the present study and the one regarding *D. repens* [38], a unique haplotype structure among isolates from Italy was found, which could be due to the geographical isolation of this area: the Italian Peninsula enclosed by the Alps.

Furthermore, the majority of the Italian sequences (16/17) formed three unique haplotypes (Di2, Di5, Di9). In the study on *D. repens* [38], sequences from Italy also formed three unique *D. repens* haplotypes. Based on these results, it can be concluded that *Dirofilaria* spp. from Italy are segregated from other European countries and the spread of Italian haplotypes to neighbouring countries is very limited, especially compared to evidence of shared haplotypes in central Europe (i.e. *D. immitis* in Romania and Hungary) or in the Baltic region (*D. repens*; [38]).

Two unique haplotypes (Di4, Di7) encompassed only Slovakian sequences (*n* = 5); however, the relatively low number of isolates from this country was examined. Slovakia has become endemic for *D. immitis* during the last 5 years. Nowadays, heartworm disease as a mono-infection or as a co-infection with *D. repens* represents 45% of all dirofilarial infections diagnosed in the endemic regions situated in the south-western part of the country [51]. Furthermore, *D. immitis* infections have been documented in local dogs in Slovakia since 2005 giving the parasite time to assert its unique genetic variation [52]. Our previous study on *D. repens* [38] successfully delineated 18 haplotypes among 95 isolates from 10 countries based on the same markers also used in this study (total sequence length of 1520 bp). Although the current study involved more isolates (*N* = 122) and a longer mt sequence (1721 bp), it still yielded fewer haplotypes and also fewer variable sites in the full-length sequences. Following the elimination of haplotypes from non-European countries, *D. repens* still manifested higher genetic diversity: 17 haplotypes versus nine *D. immitis* haplotypes. The overall nucleotide diversity and haplotype diversity for both species were low. Thus, it can be interpreted that *D. repens* has higher genetic diversity than *D. immitis* and/or *D. immitis* is more conserved in the targeted markers.

As mentioned previously, a study based on the *D. immitis* sequences available in the GenBank successfully identified five haplotypes of *D. immitis* and 12 haplotypes of *D. repens* by targeting the COI and 12S rDNA genes which supports our main finding that *D. immitis* has lower genetic diversity than *D. repens* [47].

Contrarily, in the state of Florida in the USA, 11 haplotypes were delineated among 25 *D. immitis* isolates originating from free-ranging coyotes from 28 counties by using the Pgp gene (P-glycoprotein) [53]. This may suggest that the genetic diversity of *D. immitis* might be higher based on genes other than (mt) DNA.

A limitation of the study design could be the problematic identification of haplotypes from microfilariae in blood from dogs co-infected with different haplotypes of *D. immitis*. However, we believe this limitation did not occur in our work since the sequencing of microfilaria of all blood isolates showed no nucleotide paralogs in obtained chromatograms, which supports that all isolates belonged to an individual lineage rather than a mix of different lineages of microfilaria within the same host. Yet, it was observed that the sequences obtained from microfilaria appear to have higher genetic diversity than the adult worm isolates since sequences obtained from MF were encompassed in seven unique haplotypes while almost all of the sequences obtained from adult worms were encompassed in the most common haplotype Di1.

The phylogenetic analysis for both fragments of the COI gene supported the results obtained from the median spanning network analysis (haplotype network). All studied sequences appear to be closely related in the phylogenetic trees except for the Italian sequences, which appear to form an individual clade, further supporting the idea that the Italian sequences are genetically different and geographically segregated.

The last phylogenetic analysis based on the NADH gene showed only one major clade that included all sequences from this study and obtained sequences from the GenBank from Australia and China. This result is similar to the output of phylogenetic analysis of *D. repens* NADH gene, which revealed that most sequences from that study grouped together with numerous sequences of *D. repens* obtained from the GenBank [38].

It seems that the NADH gene is highly conserved in the *Dirofilaria* spp.; also in the previous study on *D. repens* we observed low genetic diversity in this gene [38].

## Conclusions

This study shows that there is an association between the genetic diversity and geographic origin of the *D. immitis* isolates when analysing (mt) DNA markers (COI, NADH), as was observed in the sequences from Italy, Hungary, Romania and Slovakia. *Dirofilaria immitis* is more conserved than *D. repens* in the mt markers COI and NADH by having half the number of haplotypes. NADH is a conserved mt gene in *Dirofilaria* spp. regardless of the geographic origin of the isolates.

### Supplementary Information

**Additional file 1: Dataset S1**. List of isolates, assigned haplotypes and their accession numbers.

## Data Availability

Not applicable.
